# A study of ventilator-associated pneumonia: Incidence, outcome, risk factors and measures to be taken for prevention

**DOI:** 10.4103/0019-5049.72643

**Published:** 2010

**Authors:** Hina Gadani, Arun Vyas, Akhya Kumar Kar

**Affiliations:** Department of Anaesthesiology, M P Shah Medical College, Jamnagar, Gujarat - 361 008, India

**Keywords:** Intensive care unit, mechanical ventilation, ventilator-associated pneumonia

## Abstract

Ventilator-associated pneumonia (VAP) is a major cause of hospital morbidity and mortality despite recent advances in diagnosis and accuracy of management. However, as taught in medical science, prevention is better than cure is probably more appropriate as concerned to VAP because of the fact that it is a well preventable disease and a proper approach decreases the hospital stay, cost, morbidity and mortality. The aim of the study is to critically review the incidence and outcome, identify various risk factors and conclude specific measures that should be undertaken to prevent VAP. We studied 100 patients randomly, kept on ventilatory support for more than 48 h. After excluding those who developed pneumonia within 48 h, VAP was diagnosed when a score of ≥6 was obtained in the clinical pulmonary infection scoring system having six variables and a maximum score of 12. After evaluating, the data were subjected to univariate analysis using the chi-square test. The level of significance was set at *P*<0.05. It was found that 37 patients developed VAP. The risk factor significantly associated with VAP in our study was found to be duration of ventilator support, reintubation, supine position, advanced age and altered consciousness. Declining ratio of partial pressure to inspired fraction of oxygen (PaO_2_/FiO_2_ ratio) was found to be the earliest indicator of VAP. The most common organism isolated in our institution was Pseudomonas. The incidence of early-onset VAP (within 96 h) was found to be 27% while the late-onset type (>96 h) was 73%. Late-onset VAP had poor prognosis in terms of mortality (66%) as compared to the early-onset type (20%). The mortality of patients of the non-VAP group was found to be 41% while that of VAP patients was 54%. Targeted strategies aimed at preventing VAP should be implemented to improve patient outcome and reduce length of intensive care unit stay and costs. Above all, everyone of the critical care unit should understand the factors that place the patients at risk of VAP and utmost importance must be given to prevent VAP.

## INTRODUCTION

Ventilator-associated pneumonia (VAP) refers to bacterial pneumonia developed in patients who have been mechanically ventilated for a duration of more than 48 h.[1] It ranges from 6 to 52% and can reach 76% in some specific settings.[2] Hospital-acquired pneumonia (HAP) is the pneumonia after 48 h or more after admission, which did not appear to be incubating at the time of admission. The presence of HAP increases hospital stay by an average of 7–9 days per patient[3,4] also imposes an extra financial burden to the hospital. The risk of VAP is highest early in the course of hospital stay, and is estimated to be 3%/day during the first 5 days of ventilation, 2%/day during days 5–10 of ventilation and 1%/day after this.[5]

Lack of a gold standard for diagnosis is the major culprit of poor outcome of VAP. The clinical diagnosis based on purulent sputum may follow intubation or oropharyngeal secretion leakage around airway, chest X-ray changes suspected of VAP may also be a feature of pulmonary oedema, pulmonary infarction, atelectasis or acute respiratory distress syndrome. Fever and leukocytosis are non-specific and can be caused by any condition that releases cytokines. Although microbiology helps in diagnosis, it is not devoid of pitfalls. In fact, it was proven that colonization of airway is common and presence of pathogens in tracheal secretions in the absence of clinical findings does not suggest VAP.[6,7] The Clinical Pulmonary Infection Scoring (CPIS) system originally proposed by Pugin and others helps in diagnosing VAP with better sensitivity (72%) and specificity (80%). This study aims to critically review the incidence and outcome, identify various risk factors and to conclude specific measures that should be undertaken to prevent VAP.

## METHODS

The study was conducted over a period of 1.5 years, extending from July 2008 to December 2009, in an intensive care unit (ICU) of a tertiary care centre. A total of 100 patients who were kept on mechanical ventilator were randomly selected. Cases included were patients of both sexes who were kept on mechanical ventilator for more than 48 h, having the age of >15 years. Patients who died or developed pneumonia within 48 h or those who were admitted with pneumonia at the time of admission and patients of ARDS (Acute Respiratory Distress Syndrome) were excluded from the study. Most of the patients put of ventilator support were primarily treated elsewhere with antibiotics either in the indoor ward or in other health care centres that was not traceable. A questionnaire was prepared and each patient selected to be included in the study was screened and monitored according to the questionnaire. Age, sex, date of admission to ICU, date of initiating mechanical ventilation and mode of assess to the patients’ airway, i.e. orotracheal or tracheostomy, were recorded. Indication of mechanical ventilation was noted. In each patient, ventilator mode and settings were recorded and any change in setting was recorded daily. Patients’ vitals, general and physical examination, oxygen saturation and position of the patients were monitored regularly. During the initial stage of ventilation, patients were adequately sedated. All necessary measures were taken for prevention of hospital-acquired infections. A battery of routine investigations was performed and special investigations, like culture of tracheal tube, blood and urine and others like serum cholinesterase levels when needed, were performed. Sputum from the patients were collected from the tip of the suction catheter and transported to the laboratory in a sterile tube. Patients were monitored from the date of inclusion in the study to the final outcome in the ICU. VAP was diagnosed on clinical grounds based on the modified CPIS system [Table 1] originally developed by Pugin and others,[1] giving 0–2 points each for fever, leukocyte count, oxygenation status, quantity and purulence of tracheal secretions, type of radiographic abnormality and result of sputum culture and Gram stain. The VAP group was classified into two groups, early-onset type (within 48–96 h) and late-onset type (>96 h). Once the clinical suspicion was established, empirical antibiotic therapy was initiated based on guidelines prescribed by the American Thoracic Society. Patients were routinely screened by arterial blood gas (ABG) analysis every 12 hourly and appropriate steps were taken to correct any change.

**Table 1:** Clinical pulmonary infection scoring system

CPIS points	0	1	2
Tracheal secretions	Rare	Abundant	Purulent
Leukocyte count (mm^3^)	>4,000 and <11,000	<4,000 and >11,000	<4,000 or >11,000 + band forms
Temperature (°C)	>36.5 and <38.4	>38.5 and <38.9	>39 or <36
PaO_2_/FIO_2_ ratio (mmHg)	>240 or ARDS	-	≤240 and no ARDS
Chest radiograph	No infiltrate	Diffuse infiltrate	Localized infiltrate
Culture of tracheal aspirate	Negative	-	Positive

CPIS: Clinical pulmonary infection scoring

### Statistical analysis

The study cohort was classified into two groups, “early-onset VAP (onset after 48 h but within 96 h)” and “late-onset VAP (onset after 96 h)”. With a sample size of 70, calculated based the results of a previous study,[8] the power of the study was set at 80%. After evaluating, the data were subjected to univariate analysis using the chi-square test. The level of significance was set at *P*<0.05.

## RESULTS

The study cohort comprised of 100 patients of various cases of poisoning, neurological disorders, sepsis and others. The mean age of the patients was 34 years, having a predominance of male population. Of the 100 patients, 37 patients developed VAP during the ICU stay. The mean duration of mechanical ventilation was found to be 11 days for the non-VAP group and 19 days for the VAP group. It was analysed in our study that those requiring prolonged ventilator support (>15 days) had a significantly higher incidence of VAP (*P*-value, 0.001). Supine position and stuporous, comatose patients were found to be risk factors, having a high incidence of VAP, and proved to be statistically significant (*P*-value, 0.003 and 0.0023, respectively). The PaO_2_/FiO_2_ ratio was analysed in VAP patients and was found to be <240 mmHg in 86% of the cases. In the remaining 14%, the ratio was higher (>240 mmHg). Of the 37 patients who developed VAP, 10 patients developed early-onset (27.02%) VAP and 27 patients developed the late-onset type (72.97%). The overall mortality was found to be 46%, while mortality in the VAP patients was found to be 54%. The mortality of the early-onset type was found to be 20%. In case of the late-onset type, it was found to be 66.67%. Late-onset VAP had a significantly high association (*P*=0.0234) as far as mortality was concerned in comparison with early-onset pneumonia. The order of prevalence of organism in our study was found to be Pseudomonas (43.2%), *Klebsiella* (18.91%), followed by MRSA, *E. coli*, Acinetobacter, MSSA and *S. pneumoniae*.

## DISCUSSION

In the study of our set up, males predominated (62%). Although the incidence of VAP was also high in males, it was statistically not significant (*P*=0.2086) [Table 2]. The mean age group in our study was 34 years. The young population group in our set up is due to the number of cases of poisoning that predominated our study.

**Table 2:** Gender distribution

Gender	No. of cases	No. of VAP cases	Percentage of VAP cases	*P*-value
Male	62	26	41.93	0.2086*
Female	38	11	28.97	
Total	100	37		

* *P*>0.05, not significant,

VAP: Ventilator-associated pneumonia

The incidence of VAP in our setting was 37%. In the era of advanced diagnosis and early management of possible complications, the incidence tends to be lower. In recent studies,[9,10] the reported incidence is very low, ranging from 15 to 30%. The high incidence in our study may be due to a lower number of cases (i.e., 100) and lack of adequate nursing staff (which should ideally be 1:1 as compared to 4:1 in our institute) which may have adversely affected the quality of care given to the patients. Another factor in our study was a higher number of cases of patients of Organo-phosphorous poisoning that required prolonged ventilation, which is proved to be a risk factor having a statistically significant relation (*P*-value, 0.001; Table 3) with incidence of VAP, and may have influenced the incidence. The mean duration of ventilation in our study for non-VAP patients was 11 days whereas it was almost 19 days for VAP patients, which almost matches other studies.[11] It was proved in our study that duration of mechanical ventilation is an important risk factor for VAP, which is similar to other studies[12] where the mean duration of ventilation was around 10 days and the incidence of VAP was found to be 9.3%.

**Table 3:** Comparison of duration of mechanical ventilation and incidence of VAP ventilator-associated pneumonia

Days on ventilator	Cases	VAP	Percentage	*P*-value
≤15	84	25	29.76	0.001**
>15	16	12	75	
Total	100	37		

** *P*<0.01, highly significant,

VAP: Ventilator-associated pneumonia

The mean duration of ventilation can effectively be reduced by administrating a proper weaning protocol. It was estimated that 42% of the time a medical patient spends on the mechanical ventilator is during the weaning process.[13] Among the various methods of weaning, spontaneous breath trial has been proved to be very effective as compared with the intermittent mandatory ventilation (IMV) because of the fact that IMV promotes respiratory fatigue.[14,15] A once-daily trial of spontaneous breathing and a prolonged period of rest may be the most effective methods of weaning to recondition respiratory muscles that may have been weakened during mechanical ventilation.[16,17]

Reintubation resulted in a very high incidence of VAP[18] and proved to be an independent risk factor in various studies.[19] This may be due to impaired reflexes after prolonged intubation or due to the altered level of consciousness, increasing the risk of aspiration. In our study, the number of patients reintubated were only five in number, but four patients developed VAP. This number is too less to compare with other studies. A recent case–control study of 135 patients following heart surgery also found reintubation to be a major risk factor as VAP occurred in 92% of the reintubated patients versus 12% of the control subjects.[20]

It was noted that patients having unaffected lung during admission, like snake bite and meningitis, have a considerably low incidence of VAP and a significantly higher incidence of VAP in supine positioning as compared with the semi-recumbent position (*P*=0.003) [Table 4] because it may facilitate aspiration, which may be decreased by a semi-recumbent positioning matches to the outcome of the other studies when position is considered as a risk factor.[21–23] In fact, it was proved using radioactive-labeled enteral feeding, that cumulative numbers of endotracheal counts were higher when patients were placed in the completely supine position (0°) as compared with a semi-recumbent position (45°).[21,22] Infection in patients in the supine position was strongly associated with the simultaneous administration of enteral nutrition. Thus, intubated patients should be managed in a semi-recumbent position, particularly during feeding.

**Table 4:** Incidence of VAP in supine vs. semi-recumbent position

	Total no. of cases	VAP	Percentage	*P*-value
Supine	30	18	60	0.003**
Semi-recumbent	70	19	27.14	
Total	100	37		

** *P*<0.01, highly significant,

VAP: Ventilator-associated pneumonia

Level of consciousness has a significant impact on the incidence of VAP. It was found in our study that the incidence of VAP in stuporous (62.5%) and comatose (50%) patients is significantly higher (*P*=0.0023) than that in conscious (35.75%) and drowsy (18.42%) patients [Table 5]. This may be due to the higher chances of aspiration in comatose patients. An early and planned tracheostomy was found to decrease the VAP significantly but could not be studied as it will take time to be accepted by one and all. When used for stress ulcer prophylaxis, Sucralfate appears to have a small protective effect against VAP because it does not raise the gastric pH like H_2_ receptor antagonists.[11] Therefore, whenever feasible, Sucralfate should be used instead of H_2_ receptor antagonists.

**Table 5:** Relation of VAP with consciousness status

Consciousness status	Frequency	Developed VAP	Percentage	*P*-value
Conscious and drowsy	66	17	25.75	0.0023**
Stuporous and comatose	34	20	58.82	
Total	100	37		

** *P*-value <0.01, highly significant,

VAP: Ventilator-associated pneumonia

The PaO_2_/FiO_2_ ratio was assessed during the course of ventilatory support and it was observed that the ratio dropped at least 12–24 h before the onset of the clinicoradiologic picture suggestive of VAP. Thus, a decline in the PaO_2_/FiO_2_ ratio was found to be an early indicator of onset of VAP. Although fever was found almost in 100% of the patients, it was of no significance as far as diagnosis was concerned because most of the patients were of poisoning cases that required atropine in due course of time and, as such, fever is a non-specific sign itself.

The most common organism associated with VAP is Pseudomonas (43.24%), followed by *Klebsiella* (18.91%). Also, the overall mortality rate was high in the Pseudomonas group (62.5%). In other studies,[24,25] isolation of Pseudomonas ranges from 15 to 25%. Susceptibility testing could not be studied in all patients due to a lack of clinical microbiologic support as it is not done routinely and sending samples outside is not allowed by the hospital authority except in special cases.

Early-onset VAP in our study was found to be 27.02% while in various study it was found to be around 40%.[26] The low incidence in our study may be due to antibiotic use before admission to the ICU. Studies[27] have shown that previous antibiotic use decreases early-onset VAP but markedly increases multidrug-resistant (MDR) pathogens, which is also reflected in our study. Our study also demonstrated that early-onset VAP had a good prognosis as compared with the late-onset type in terms of mortality, which is also statistically significant (*P*=0.0234) [Table 6]. Probably, the de-escaltation strategy[28] fully endorsed by the American Thoracic Society, which means initiation of a broad-spectrum antibiotic and changing to a narrow spectrum after the sensitivity results are made available, will reduce inappropriate antibiotic use and, subsequently, the drug-resistant pathogens. Although it is not performed in our ICU, invasive bronchoscopic sample collection and quantitative sample culture reduces inappropriate antibiotic use.[29]

**Table 6:** Outcome of patients of ventilator-associated pneumonia

Outcome	Early-onset VAP	Late-onset VAP	Total VAP	*P*-value	Non-VAP	*P*-value
Expired	2	18	20	0.0234*	26	0.2988**
Survived	8	09	17		37	
Total	10	27	37		63	

* *P*<0.05, significant;

** *P*-value >0.05, not significant,

VAP: Ventilator-associated pneumonia

The mortality rate in our study was found to be 54.05% in the VAP group as compared to 41.2% in the non-VAP group. Although the incidence was slightly high in VAP, it is statistically not significant. Table 6 implies that VAP as such does not increase the mortality in ICU patients.

Hand washing is widely recognized as an important but underused measure to prevent nosocomial infections.[30] According to the 2004 CDC (Center for Disease Control) guidelines, hands should be washed before and after patient contact and also in between patient contact. Chlorhexidine has been shown to be effective in the control of ventilator-circuit colonization and pneumonia caused by antibiotic-resistant bacteria.[31] Oropharyngeal decontamination with Chlorhexidine solution has also been shown to reduce the occurrence of VAP in patients undergoing cardiac surgery.[32]

## CONCLUSION

We arrive at the following conclusions:

- Incidence is directly proportional to duration of mechanical ventilation and re-intubation is a strong risk factor for development of VAP. Therefore, duration of ventilation has to be reduced to get rid of morbidity and mortality associated with mechanical ventilation, which can be achieved by administering a proper weaning protocol and titrating sedation regimens as per the need of the patients.
- Promoting nasogastric feeding. Although necessary for critically ill patients, it should be given keeping the patients in a semi-recumbent position with the head end elevated to 45° because the supine position promotes aspiration.
- A decrease in the PaO_2_/FiO_2_ ratio is an early predictor of VAP.
- Pseudomonas is the most common organism in our institution.
- Late-onset VAP is associated with poor prognosis as compared to the early-onset variety.
- Inappropriate antibiotic use prior to ventilatory support decreases the early-onset variety but predisposes to a high incidence of MDR pathogens.
